# Incidentally discovered urothelial papilloma in a 9-year-old girl

**DOI:** 10.1016/j.eucr.2026.103421

**Published:** 2026-03-28

**Authors:** Farzaneh Sharifiaghdas, Mohsen Firoozi, Seyedhossein Rabani, Arash Ranjbar, Behnam Dejman, Ala Torabi

**Affiliations:** a Department of Urology, Urology and Nephrology Research Center, Shahid Labbafinejad Medical Center, Shahid Beheshti University of Medical Sciences, Tehran, Iran; b Fellowship of Pediatric Urology, Department of Urology, Shahid Labbafinejad Medical Center, School of Medicine, Shahid Beheshti University of Medical Sciences, Tehran, Iran; c Urology and Nephrology Research Center, Shahid Labbafinejad Medical Center, Shahid Beheshti University of Medical Sciences, Tehran, Iran; d Department of Radiology, Shariati Hospital, Tehran University of Medical Science, Iran

**Keywords:** Urothelial papilloma, Pediatric bladder tumor, Incidental finding, Transurethral resection

## Abstract

Urothelial papilloma is a rare benign bladder tumor in children, usually presenting with hematuria. We report a 9-year-old girl with incidentally discovered bladder urothelial papilloma during evaluation of abdominal pain, without urinary symptoms. Ultrasound revealed a small papillary bladder lesion, which was completely resected via transurethral resection. Histopathology confirmed urothelial papilloma. This case highlights the importance of considering bladder pathology even in asymptomatic pediatric patients. Complete resection is curative, but careful follow-up is recommended due to probability of recurrence, although uncommon.

## Highlights

• Urothelial papilloma of the bladder is extremely rare in the pediatric population.
• Most pediatric bladder tumors present with hematuria; incidental detection is unusual.
• Ultrasound is a valuable non-invasive tool for initial evaluation
• Complete transurethral resection of urothelial papilloma is curative without need for adjuvant therapy.
• Long-term surveillance is recommended despite excellent prognosis.

## 1 Introduction

Bladder tumors are rare in the pediatric population, accounting for less than 1% of all bladder neoplasms. The majority of urothelial bladder tumors in children are low-grade papillary lesions, including urothelial papilloma (UP) and papillary urothelial neoplasm of low malignant potential (PUNLMP).^1^^,^^2^ UP is a benign tumor with an excellent prognosis, though it is extremely uncommon in children. Hematuria is typically the main presenting symptom, but in rare cases, lesions may be discovered incidentally during imaging for unrelated abdominal complaints.^1^

## 2 Case presentation

A 9-year-old girl was referred for evaluation of mild, intermittent abdominal pain lasting two months. She had no urinary symptoms such as dysuria, urgency, or hematuria. Physical examination and laboratory tests were normal. Abdominal ultrasound revealed a 16 × 15 mm echogenic lesion in the right lateral bladder wall with vascularity and small calcifications. There was mild right hydronephrosis.

Cystoscopy demonstrated a solitary, frond-like papillary tumor near the right ureteral orifice. Transurethral resection of the bladder tumor (TURBT) was performed by the pediatric resectoscope sheath under general anesthesia. The tumor was resected completely with clear margins. Grossly, the tissue fragments were soft and cream-colored (Fig. 1 video1). Microscopically, the lesion consisted of delicate fibrovascular stalks lined by normal-appearing urothelial cells without atypia or invasion (Fig. 2). These findings confirmed a diagnosis of urothelial papilloma. Postoperative recovery was uneventful, and the patient remains asymptomatic and recurrence-free one year postoperatively.

**Fig. 1:**
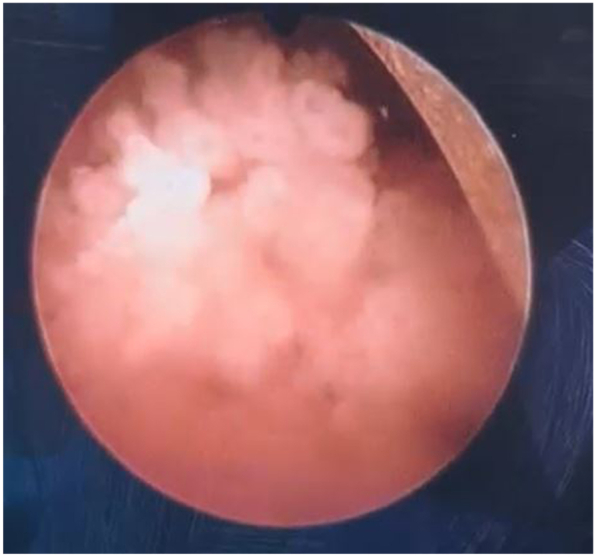
Endoscope shows frond-like papillary masses near the right ureteral orifice.

**Fig. 2:**
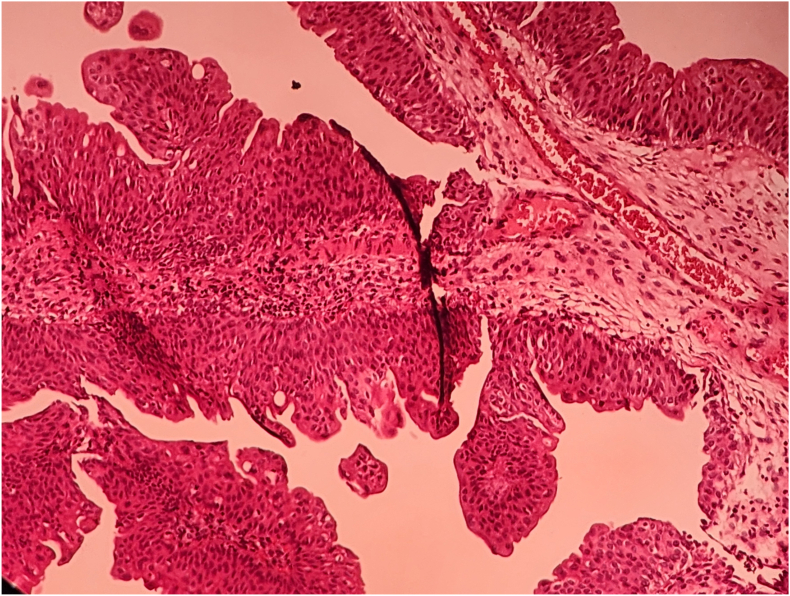
Photomicrographs image demonstrates papillary structures with fibrovascular cores and normal urotheilial lining and umbrella cell layer.

## 3 Discussion

Urothelial papilloma of the bladder is an extremely rare benign tumor in children. Most pediatric bladder tumors present with painless hematuria, making this incidental finding in a child without urinary symptoms unusual. Ultrasound is the preferred initial imaging modality for pediatric urinary tract evaluation, providing a non-invasive way to detect intravesical masses. In this case, the mass was found during investigation for unrelated abdominal pain.^1^, ^2^, ^3^.

Differential diagnoses of pediatric bladder masses include embryonal rhabdomyosarcoma, PUNLMP, inflammatory myofibroblastic tumor, and inverted papilloma.^4^

Rhabdomyosarcoma is the most common malignant bladder tumor in children and typically presents as a large, infiltrative, grape-like mass causing urinary obstruction. PUNLMP is histologically similar to UP but shows minimal thickening or atypia. Inflammatory myofibroblastic tumors are spindle-cell proliferations often associated with inflammation and ALK rearrangements, while inverted papilloma shows endophytic rather than exophytic growth. Careful histopathological evaluation distinguishes these entities.^4^^,^^5^

Complete transurethral resection is curative for UP. Intravesical chemotherapy or immunotherapy is unnecessary. Follow-up is important because, although recurrence is rare, it has been reported in a few pediatric cases. Regular ultrasonographic surveillance is recommended every 3–6 months during the first three years and annually thereafter. Prognosis is excellent, with no documented cases of malignant transformation in children.^1^^,^^6^

## 4 Conclusion

Due to the limited number of reported pediatric urothelial papilloma cases, our current knowledge regarding recurrence rates and long-term prognosis remains incomplete. Therefore, close follow-up with periodic cystoscopy and ultrasound is recommended to ensure early detection of any recurrence. Further case reports and multicenter studies are needed to improve understanding of the biological behavior and optimal surveillance strategies for this rare pediatric condition.

## Availability of data and material & code availability

Not applicable.

## CRediT authorship contribution statement

**Farzaneh Sharifiaghdas:** Conceptualization, Supervision. **Mohsen Firoozi:** Conceptualization, Data curation. **Seyedhossein Rabani:** Validation, Writing – original draft, Writing – review & editing. **Arash Ranjbar:** Conceptualization. **Behnam Dejman:** Conceptualization, Data curation. **Ala Torabi:** Validation, Writing – original draft, Writing – review & editing.

## Ethics approval & consent to participate & consent for publication

Parents of the patient in this case report signed informed consent. The context of the consent forms included that the patient's images and clinical information would be reported in a journal without mentioning his/her name.

## Funding

No funding was received.

## Conflicts of interest

The authors declare no conflicts of interest.
